# Evaluation of Race-Neutral Glomerular Filtration Rate Estimating Equations in an Indian Population

**DOI:** 10.1016/j.ekir.2024.09.020

**Published:** 2024-10-10

**Authors:** Ashok Kumar Yadav, Jaskiran Kaur, Prabhjot Kaur, Kajal Kamboj, Yoshinari Yasuda, Masaru Horio, Arnab Pal, Nusrat Shafiq, Nancy Sahni, Harbir Singh Kohli, Seiichi Matsuo, Vivek Kumar, Vivekanand Jha

**Affiliations:** 1 Department of Experimental Medicine and Biotechnology, Postgraduate Institute of Medical Education and Research, Chandigarh, India; 2 Department of Nephrology, Postgraduate Institute of Medical Education and Research, Chandigarh, India; 3 Department of Nephrology, Nagoya University Graduate School of Medicine, Nagoya, Japan; 4 Department of CKD Initiatives, Nagoya University Graduate School of Medicine, Nagoya, Japan; 5 Division of Health Sciences, Graduate School of Medicine, Osaka University, Osaka, Japan; 6 Department of Biochemistry, Postgraduate Institute of Medical Education and Research, Chandigarh, India; 7 Department of Pharmacology, Postgraduate Institute of Medical Education and Research, Chandigarh, India; 8 Department of Dietetics, Postgraduate Institute of Medical Education and Research, Chandigarh, India; 9 The George Institute for Global Health, New Delhi; 10 School of Public Health, Imperial College, London, UK; 11 Manipal Academy of Higher Education, Manipal, India

**Keywords:** CKD, eGFR, inulin, iohexol, race free eGFR

## Abstract

**Introduction:**

Glomerular filtration rate (GFR) estimation equations have not been extensively validated in the Indian population. Preliminary data showed that the widely used creatinine-based Chronic Kidney Disease Epidemiology Collaboration (CKD-EPI_Cr_) 2009 significantly overestimated GFR in Indians. Newer estimated GFR (eGFR) equations based on creatinine and cystatin C, omitting the race, have been recently proposed. We investigated the performance of race-free eGFR equations in the Indian population.

**Methods:**

Patients with chronic kidney disease (CKD) and potential kidney donors attending the outpatient clinic at the Postgraduate Institute of Medical Education and Research Chandigarh, India, were screened for enrolment. GFR was measured by urinary clearance of inulin and plasma clearance of iohexol. Performance of eGFR equations (CKD-EPI_Cr(2021)_, CKD-EPI_Cr-Cys(2021),_ CKD-EPI_Cr(2009)_, CKD-EPI_Cr-Cys(2012)_, CKD-EPI_Cys_, 2020_Csy-B2M-BTP_ and 2020_Cr-Csy-B2M-BTP_, EKFC_cr,_ EKFC_cys_, and EKFC_cr-cys_) were assessed against measured GFR (mGFR) using bias, precision, and accuracy (root mean square error [RMSE], mean absolute error [MAE] and P_30_ [% with eGFR within 30% of mGFR]).

**Results:**

A total of 412 subjects (55% with CKD), average age 47 ± 11 years with an equal distribution of males and females were enrolled. The mean mGFR in the study population was 54.2 ± 30.2 ml/min per 1.73 m^2^. The average mGFR in the potential kidney donor’s subgroup was 79.5 ± 23.2 ml/min per 1.73 m^2^. Bias was highest for creatinine-based eGFR equations (CKD-EPI_cr(2021)_: −19.2 [−21.3 to −17.0] ml/min per 1.73 m^2^and CKD-EPI_cr(2009)_: −17.0 [−19.1 to −15.0] ml/min per 1.73 m^2^). Cystatin C– (either alone or with other markers) based equations were slightly better but still did not reach P_30_ ≥ 80%.

**Conclusions:**

Race-neutral CKD-EPI_Cr(2021)_ equation did not significantly improve performance as compared to CKD-EPI_Cr(2009)_ equation. These observations emphasize the need for developing new eGFR equations for Indians and to standardize the mGFR for easy access to care providers for individualized patient care.

## Graphical abstract

**Figure:**
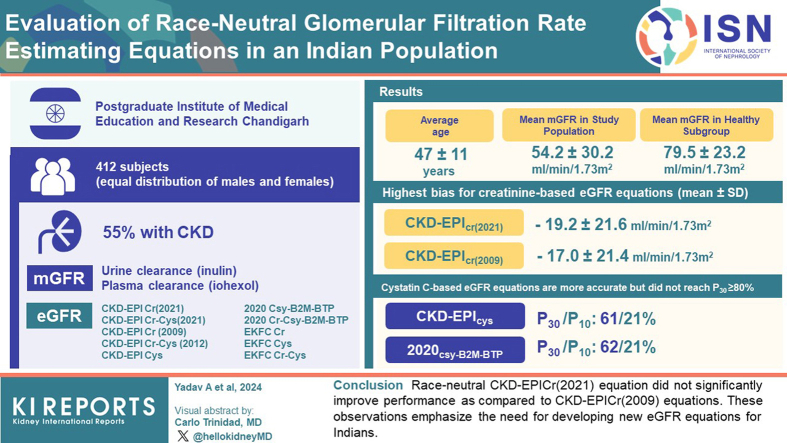

See Commentary on Page 3355

Kidney disease is recognized as a global health problem and a burden on health systems, especially in developing countries.^1^ According to the Global Burden of Disease Study, approximately 697.5 million cases of CKD were reported globally in 2017, 16.4% of whom lived in India.^2^ An accurate estimation of GFR is fundamental to the diagnosis, classification, and management of patients with kidney diseases, selection of kidney donors, and for deciding drug dosing.^3^ GFR measurement is challenging due to the complexity of the procedure involved, and its use in routine clinical practice is impractical for every patient. Fortunately, the specific value of GFR does not change management in most clinical scenarios but may be required in specific situations. GFR measurement can be protocolized and implemented in clinical care as required. GFR estimating equations have been developed using endogenous markers such as serum creatinine. However, the accuracy of the commonly used creatinine-based CKD-EPI_cr_ eGFR equation is poor in populations in which they were not originally developed, such as Asians or Black Africans. Given that serum creatinine is affected by non-GFR factors, such as age, gender, race, muscle mass, and dietary protein intake, eGFR equations using alternate endogenous markers such as cystatin C, and low-molecular-weight proteins such as β2-microglobulin (B2M) and β-trace protein (BTP) that may reduce the impact of non-GFR determinants have been developed.^4^

The recent controversy over the inclusion of race in eGFR equations has prompted the development of race-free equations in the USA. Recently, the CKD-EPI group refitted eGFR equations to develop race-free equations based on creatinine (CKD-EPI_cr(2021)_) and creatinine-cystatin C (eGFR CKD-EPI_cr_cys(2021)_).^5^ CKD-EPI_cr_cys(2021)_ equation without race was more accurate than those based on either creatinine or cystatin C alone and showed a smaller bias between black and non-black populations.^5^

Previously, we have shown that the creatinine-based CKD-EPI_cr(2009)_ equation overestimates mGFR in Indian subjects. Cystatin C–based equations showed less bias but could not achieve a P_30_ of 80%, which is considered an acceptable characteristic of a GFR estimating equation.^6^ Now we evaluated the performance of race-neutral CKD-EPI_cr(2021)_ and eGFR CKD-EPI_cr_cys(2021)_ equations along with CKD-EPI_cr(2009),_ CKD-EPI_cr_cys(2009),_ CKD-EPI_cys(2012),_ 2020_Csy-B2M-BTP_, 2020_Cr-Csy-B2M-BTP_, EFKC_cr_, EFKC_cys_, and EFKC_cr-cys_ versus mGFR in Indian subjects.

## Methods

### Study Setting

This study was conducted at the Postgraduate Institute of Medical Education and Research (PGIMER), Chandigarh, India. It represents data collected between 2014 and 2020 as part of studies to assess the performance of eGFR equations in the Indian population. In the initial phase (2014–2015), GFR was measured by using urinary inulin clearance. Since then, we have switched to plasma clearance of iohexol. The study protocols were approved by the Institutional Ethics Committee at the Postgraduate Institute of Medical Education and Research.

### Study Population

Adult patients diagnosed with CKD attending our outpatient clinics were screened for enrolment. Subjects from either sex, aged 18 to 70 years, and with stable clinical state for the preceding 3 months were included. We also recruited potential kidney donors. They were confirmed to be normotensive, had a normal HbA1C, eGFR (CKD-EPI_cr(2009)_)^7^ > 60 ml/min per 1.73 m^2^, and were normoalbuminuric. Subjects currently on dialysis or those who had received dialysis in the preceding 3 months; with active or past malignancy, voiding problems, urinary incontinence, allergy to inulin or iohexol or any other iodinated contrast media, limb, chronic liver disease, or heart failure with poor functional status were excluded. Subjects taking methyldopa were excluded from the urinary clearance of inulin study. All the enrolled subjects were drawn from North India, included both urban and rural inhabitants, and had provided written informed consent.

### Study Conduct

The study procedure was explained to the subjects and they were provided with a written instruction sheet for collecting 24-hour urine specimens. Study subjects reported on the day of the procedure after overnight fast of at least 8 hours. Demographic details, anthropometric measurements, diagnosis, treatment, and dietary details were recorded. Baseline blood and spot urine samples were collected before the procedure.

### Measurement of GFR

#### Urinary Inulin Clearance

**Figure 1:**
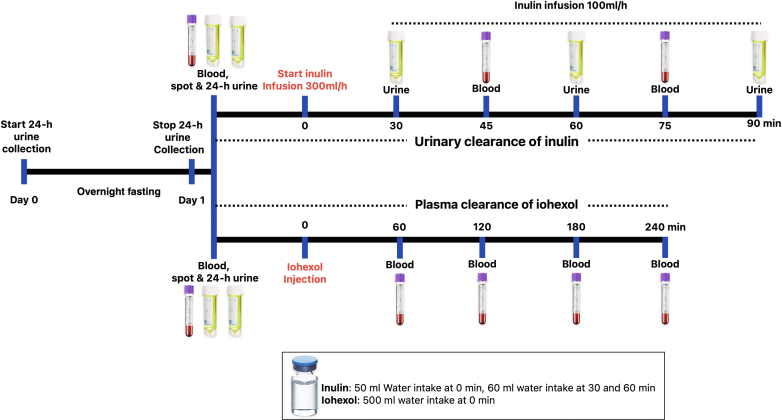
Graphical representation of methodology for sampling procedure for measurement of GFR. GFR, glomerular filtration rate.

Urinary clearance of inulin was done as described earlier (Figure 1).^6^ Briefly, subjects were orally hydrated with 500 ml of water. Baseline blood and second morning urine samples were collected. Inulin (Inulead, Fuji Yakuhin Co Ltd., Saitama, Japan) was dissolved in normal saline (10 mg/ml) and infused at 300 ml/h rate for 30 minutes, then decreased to 100 ml/h. Urine samples were obtained at 30, 60, and 90 minutes, and blood samples at 45 and 75 minutes after starting the administration of inulin. Participants were orally hydrated with 60 ml of water at 30 and 60 minutes. The inulin infusion was stopped after the last urine sample. Inulin concentrations in serum and urine samples were measured using an enzymatic method using a kit (Diacolor Inulin, Toyobo Co. Ltd., Osaka, Japan) as described earlier.^8^, ^9^, ^10^ Urinary inulin clearance was calculated as the average of 2-timed collection periods and reported as mGFR.

#### Plasma Iohexol Clearance

Participants were instructed to drink 500 ml of water before the procedure. (Omnipaque [300 mg Iodine/ml], GE Healthcare, India) was administered slowly through an i.v. route as a bolus based on the weight of the participants (5 ml for ≥50 kg and 3 ml for <50 kg). Venous blood samples were drawn from the contralateral upper limb at 60, 120, 180 and 240 minutes after the Iohexol injection (Figure 1). Plasma Iohexol was extracted using dichloromethane, and its concentrations were measured using high-performance liquid chromatography (UltiMate 3000, Thermo Fisher Scientific, USA).^11^ Iopentol was used as an internal standard to determine the percentage recovery of iohexol. External validation of the method was ensured by participation in the quarterly external quality control program (EQA iohexol, Equalis AB, Sweden) (https://www.equalis.se/en/products-services/eqa/iohexol/). Plasma clearance of iohexol was calculated per the method described by Jodal and Brochner-Mortensen and normalized for body surface area^12^ as calculated by Du Bios and Du Bios formula.^13^ The intra- and inter-assay coefficient of variation of iohexol analysis using HPLC was < 2% and <9%, respectively.

### Measurement of Serum Creatinine and Cystatin C

Serum creatinine was measured by using the Modified Jaffe’s method traceable to isotope dilution mass spectrometry standards at the central biochemistry laboratory of Postgraduate Institute of Medical Education and Research, Chandigarh, on Cobas c702 auto-analyzer (Roche Diagnostics Limited, Risch-Rotkreuz, Switzerland). Serum cystatin C was measured in the first 130 samples by colloidal gold immunoassay (Alfresa Pharma Corporation, Osaka, Japan, http://www.alfresa-pharma.co.jp) standardized for measurement of cystatin C traceable to ERM-DA471/IFCC,^14^ and in the remaining samples by Quantikine enzyme-linked immunosorbent assay kits (R&D Systems, Inc., Minneapolis, MN) correlated to cystatin C reference standard supplied by the Joint Research Centre Institute for Reference Materials and Measurements (Catalog # ERM-DA471/IFCC) with the slope of 1.07 and R^2^ of 0.998.

### B2M and BTP

B2M was analyzed using enzyme-linked immunosorbent assay (Elabsciences, USA), with an intraassay and interassay coefficient of variation of 6.63 % and 6.44%, respectively. BTP was analyzed using a sandwich enzyme immunoassay enzyme-linked immunosorbent assay kit (BioVendor - Laboratorni medicina, a.s. Karasek, Brno, Czech Republic). Intraassay and interassay coefficient of variation was 4.1 % and 4.2%, respectively.

### Other Measurements

Biochemical measurements, including urea, albumin, and hemoglobin in the blood and 24-hour urine excretions of creatinine, protein, and urea, were measured at the central biochemistry laboratory of Postgraduate Institute of Medical Education and Research, Chandigarh using Cobas c 702 auto-analyzer (Roche Diagnostic Limited, Risch-Rotkreuz, Switzerland).

Dietary protein intake was calculated using the 24-hour urine urea nitrogen method. The 24-hour urine urea nitrogen appearance was calculated as the sum of urine urea nitrogen (g/d) and non-urea nitrogen (weight [kg] × 0.031 g kg/d).^15^^,^^16^ The daily dietary protein intake was calculated by multiplying 24-hour urea nitrogen appearance multiplied by 6.25. Urine collection compliance was confirmed by using the following formula: collected urine volume/ (body weight × 21) > 0.7.^17^ For collections with values lower than 0.7, 24-hour urine creatinine excretion ≥670 mg for men or ≥450 mg for women were considered adequate.^17^

### Estimated GFR

Estimated GFR was calculated using CKD-EPI_cr(2021)_, eGFR CKD-EPI_cr_cys(2021)_, CKD-EPI_cr(2009)_, CKD-EPI_cr_cys(2012)_, CKD-EPI_cys(2012)_, 2020_Csy-B2M-BTP_, 2020_Cr-Csy-B2M-BTP_, EKFC_cr_, EKFC_cys_, and EKFC_cr-cys_ equations as listed in Supplementary Table S1.^4^^,^^5^^,^^7^^,^^18^, ^19^, ^20^

### Statistical Analyses

Data are presented as mean ± SD or median as appropriate. Categorical data were reported as frequencies or proportions. Continuous data were compared using an independent sample *t* test if the data were normal. Mann Whitney U test was used if the data were not normally distributed. Bias was computed as the difference between measured and estimated values and expressed as mean bias (95% confidence interval). Ninety-five percent distribution of bias (limits of agreement) were calculated as mean bias ± 1.96 × SD, which describes the limits that include the majority of the difference between measured and estimated values. Precision was defined as the interquartile range of differences in mGFR and eGFRs (95% confidence interval). The 95% confidence interval was calculated using bootstrapping with replacement using the percentile method (10,000 bootstraps). Accuracy was expressed as a percentage of estimated values within ± 30% and ± 10% of measured value (P_30_ and P_10_), RMSE of difference between measured and estimated values and MAE. Agreement between eGFR and mGFR was examined by plotting relative GFR differences (mGFR-eGFR) against mean of eGFR and mGFR (Bland-Altman analysis). Data were analyzed using the Statistical Package for the Social Sciences for Macintosh, version 26.0 (IBM Corp., Armonk, NY).

## Results

**Figure 2:**
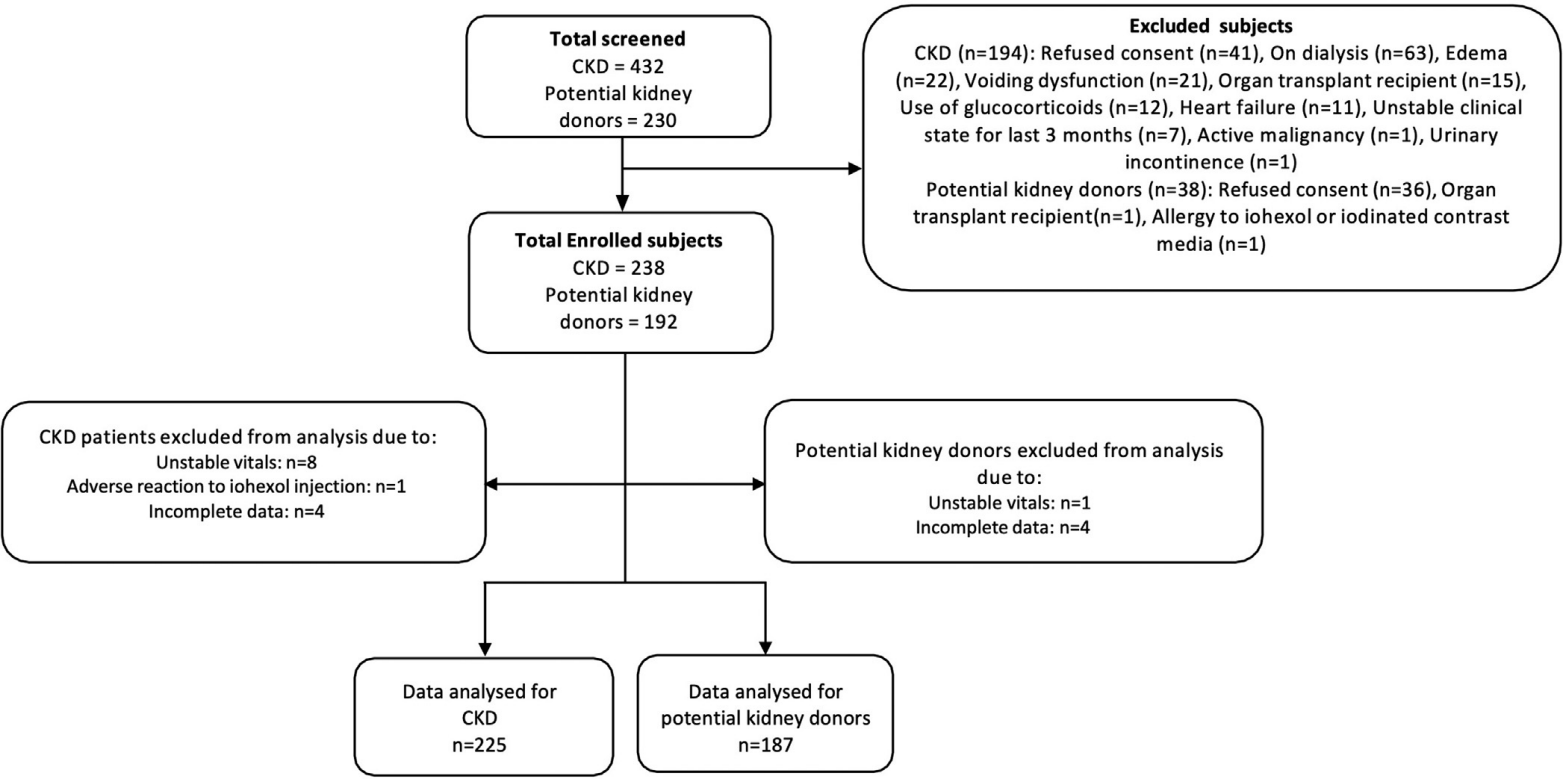
Consort flow diagram for the study participants. CKD, chronic kidney disease.

A total of 662 subjects were screened for the study, and 430 were enrolled. Eighteen more subjects were excluded (Figure 2), leaving 412 subjects, including 187 potential kidney donors and 225 subjects with CKD for the final analysis. The demographic characteristics and clinical parameters of the participants are presented in Table 1. The mean age of the population was 47 ± 11 years, with an equal distribution of males and females. About 39% were nonvegetarians. However, the frequency of meat intake was low (2.9 times per month). The mean serum creatinine was 1.45 ± 1.01 mg/dl (0.70 ± 0.31 mg/dl in potential kidney donors and 2.10 ± 0.95 mg/dl in those with CKD). The average daily protein intake was 0.74 ± 0.22 g/kg/d (0.78 ± 0.24 g/kg/d in potential kidney donors and 0.72 ± 0.20 g/kg/ d in those with CKD).

**Table 1:** Baseline demographic characteristics and clinical parameters in study population

Parameter(s)	Total study subjects (*n* = 412)	Potential kidney donors (*n* = 187)	CKD (*n* = 225)
Age (yr)	47 ± 11	46 ± 11	48 ± 12
Systolic Blood Pressure (mm Hg)	130 ± 18	124 ± 17	135 ± 18
Diastolic Blood Pressure (mm Hg)	83 ± 11	80 ± 12	85 ± 10
BMI (kg/m^2^)	25.0 ± 5.5	24.7 ± 4.4	25.3 ± 6.3
Hemoglobin (g/dl)	12.6 ± 2.0	12.5 ± 1.6	12.6 ± 2.3
Serum creatinine (mg/dl)	1.45 ± 1.01	0.67 ± 0.31	2.1 ± 0.94
Serum cystatin C (mg/l)	1.70 ± 0.72	1.08 ± 0.26	2.21 ± 0.57
Serum urea (mg/dl)	42 ± 27	22 ± 12	54 ± 25
Serum albumin (g/dl)	4.25 ± 0.38	4.39 ± 0.32	4.18 ± 0.41
24-h urine analysis			
Creatinine (mg/d)	865 (696–1113)	833 (677–1023)	931 (707–1148)
Protein (mg/d)	140 (81–384)	87 (61–131)	303 (135–1104)
Urea (mg/d)	11538 (8842–14459)	11300 (9094–14345)	11577 (8477– 14642)
Creatinine excretion (mg/d/kg)	15.4 ± 6.6	16.1 ± 7.4	14.8 ± 5.8
Protein intake			
UUN method (g/kg/d)	0.74 ± 0.22	0.78 ± 0.24	0.72 ± 0.20
Glomerular filtration rate (ml/min per 1.73 m^2^)			
mGFR	54.2 ± 30.2	79.5 ± 23.2	33.2 ± 15.9
	47.6 (28.7–74.7)	78.1 (62.5–95.0)	29.9 (20.5–42.9)
	(6, 146)	(30, 146)	(6, 100)
eGFR CKD-EPI_Cr(2021)_	73.5 ± 38.4	110.4 ± 16.4	42.8 ± 19.9
eGFR CKD-EPI_Cr-Cys(2021)_	60.8 ± 33.6	91.9 ± 21.0	35.0 ± 15.1
eGFR CKD-EPI_Cr(2009)_	71.3 ± 38.3	108.0 ± 17.7	40.7 ± 19.2
eGFR CKD-EPI_Cr-Cys(2012)_	55.1 ± 31.1	83.6 ± 20.6	31.4 ± 13.6
eGFR CKD-EPI_Cys_	50.7 ± 27.5	74.1 ± 21.7	31.2 ± 12.5
eGFR 2020_Csy-B2M-BTP_	50.8 ± 23.4	70.6 ± 18.5	34.3 ± 10.8
eGFR 2020_Cr-Csy-B2M-BTP_	58.4 ± 28.0	84.1 ± 17.2	37.0 ± 13.5
eGFR EKFC_cr_	69.2 ± 35.4	103.0 ± 16.4	41.1 ± 18.2
eGFR EKFC_cys_	54.5 ± 25.7	75.8 ± 20.0	36.7 ± 13.4
eGFR EKFC_cr-cys_	61.8 ± 29.5	89.4 ± 16.3	38.9 ± 14.5

B2M, β2-Microglobulin; BMI, body mass index; BTP, β-Trace Protein; CKD-EPI, Chronic Kidney Disease Epidemiology Collaboration; Cr, Creatinine; Cys, cystatin C; EKFC, European kidney function consortium; eGFR, estimated glomerular filtration rate; mGFR, measured glomerular filtration rate; UUN, urine urea nitrogen.

Data expressed as mean ± SD or median (25th– 75th percentile) and/or (minimum, maximum).

**Figure 3:**
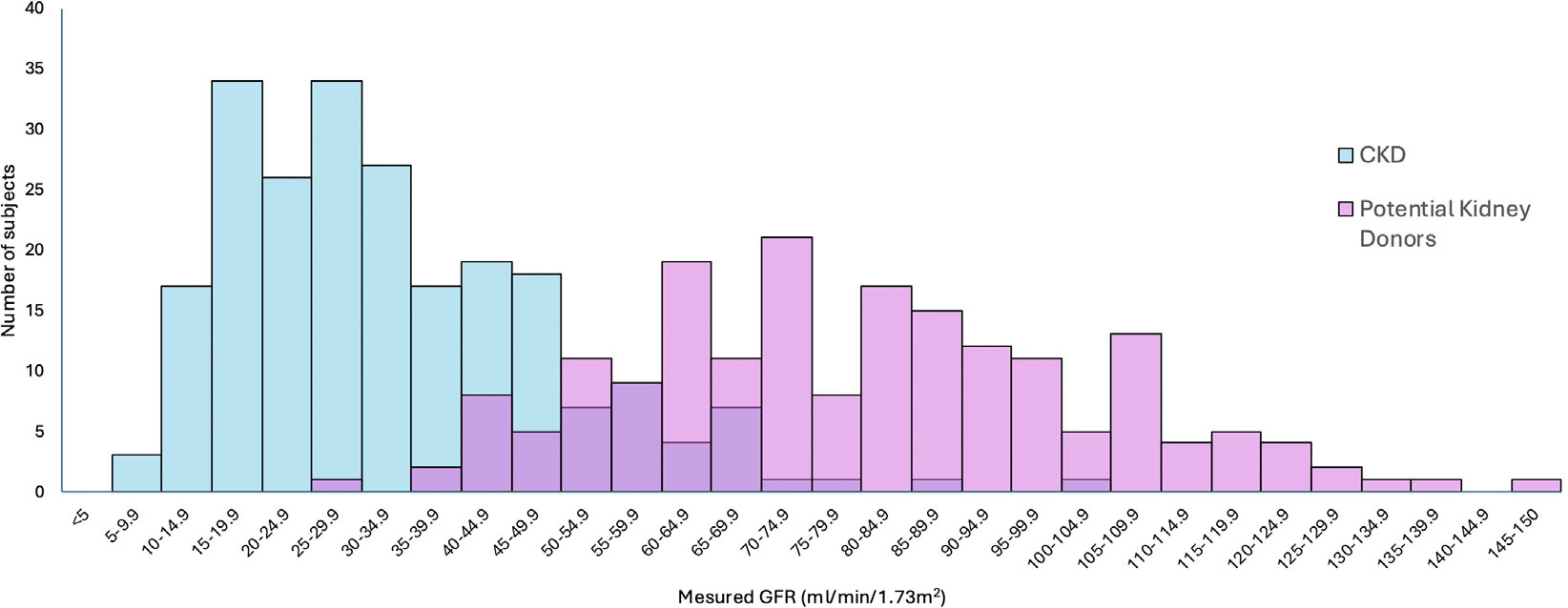
Graph showing the distribution of measured GFR in potential kidney donors and CKD subjects. Pink color for potential kidney donors, light blue color for CKD and purple color; common to both CKD and potential kidney donors. CKD, chronic kidney disease; GFR, glomerular filtration rate.

The mean mGFR of the study subjects was 54.2 ± 30.2 ml/min per 1.73 m^2^. The eGFR (in ml/min per 1.73 m^2^) was 73.5 ± 38.47 (CKD-EPI_cr(2021)_), 60.8 ± 33.6 (CKD-EPI_cr-cys(2021)_), 71.3 ± 38.3 (CKD-EPI_cr(2009)_), 55.1 ± 31.1 (CKD-EPI_cr-cys(2012)_), 50.7 ± 27.5 (CKD-EPI_cys_), 50.8 ± 23.4 (2020_Csy-B2M-BTP_), 58.4 ± 28.0 (2020_Cr-Csy-B2M-BTP_), 69.2 ± 35.4 (EKFC_cr_), 54.5 ± 25.7 (EKFC_cys_), and 61.8 ± 29.5 (EKFC_cr-cys_) (Table 1). Among the potential kidney donors, 19.8% had mGFR of < 60 ml/min per 1.73 m^2^. Of all the subjects, 247 had mGFR < 60 ml/min per 1.73 m^2^ and 165 had mGFR ³ 60 ml/min per 1.73 m^2^. In Figure 3, we present the distribution of mGFR in potential kidney donors and CKD subjects.

### Performance of the eGFR Equations

**Figure 4:**
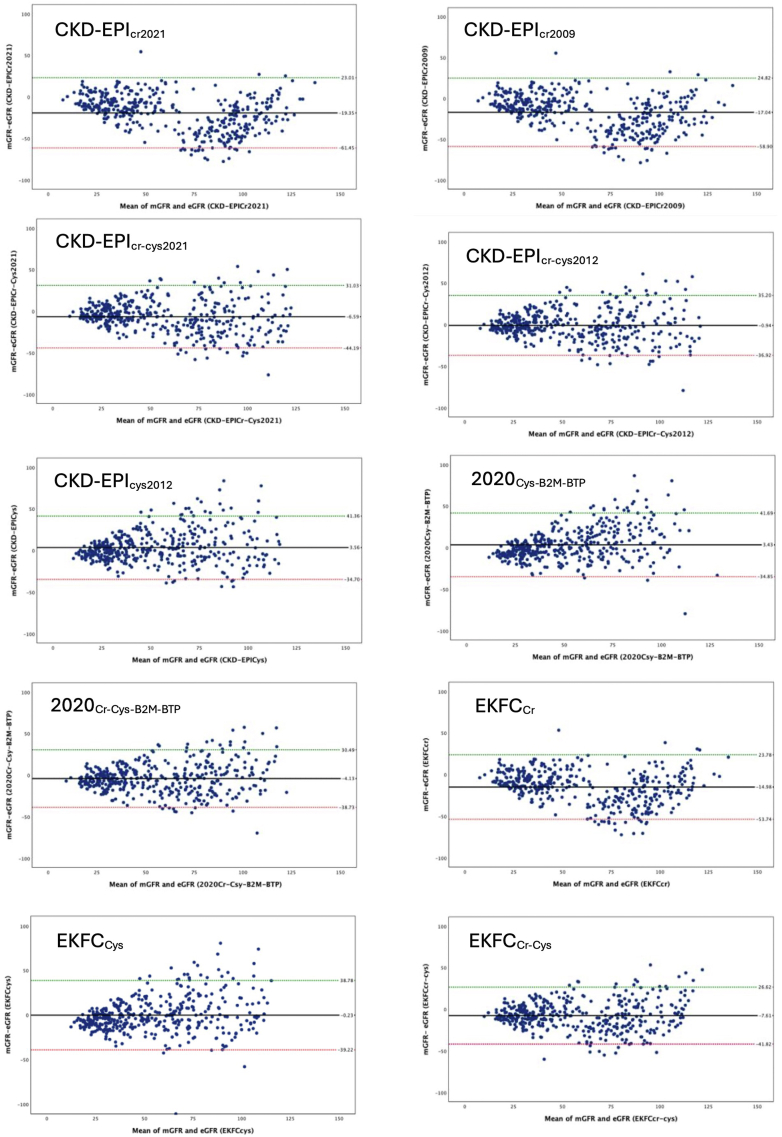
Bland-Altman plots for differences in agreement between eGFR using various estimating equations and mGFR. Three lines represent the bias (center line) and 95% limits of agreement (upper- dotted line in green and lower- dotted line in red). On Y-axis difference of measured GFR and eGFR and on X-axis, the mean of mGFR and eGFR has been plotted. eGFR, estimated glomerular filtration rate; mGFR, measured glomerular filtration rate.

**Figure 5:**
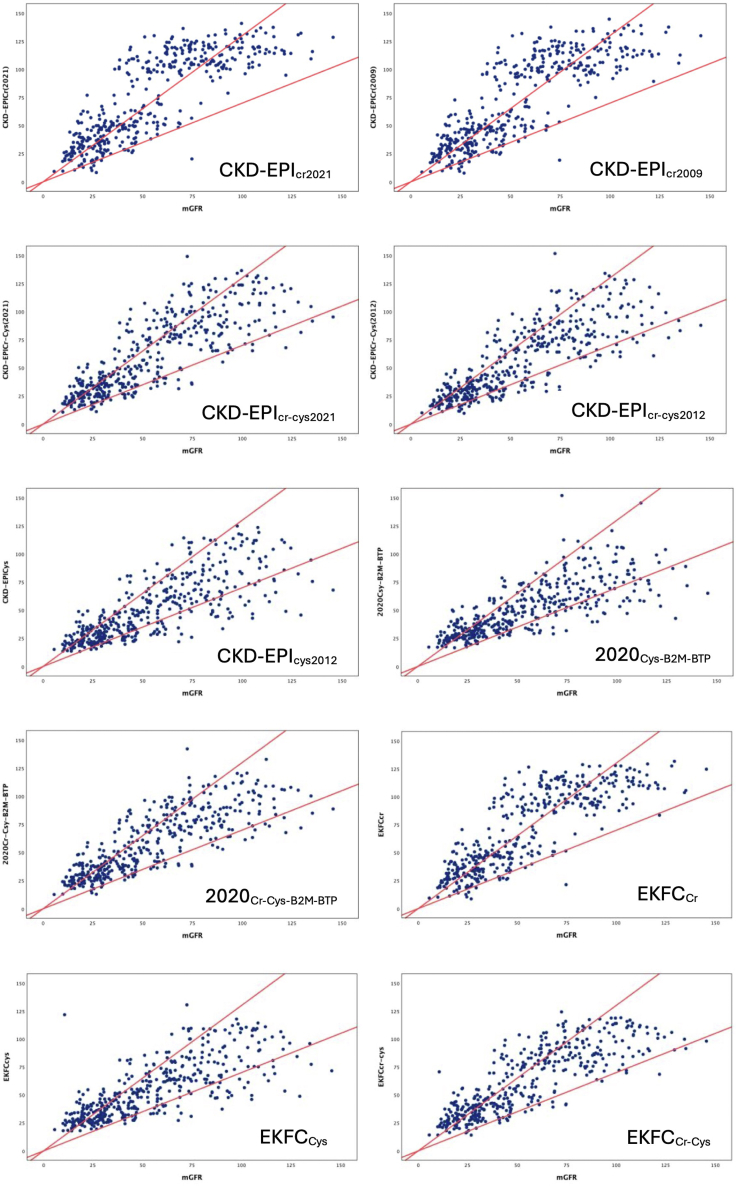
Accuracy (P_30_) of eGFR equations against mGFR. Comparison between mGFR and eGFRs. The value between diagonal lines represents the values within 30% of mGFR. eGFR, estimated glomerular filtration rate; mGFR, measured glomerular filtration rate.

In Table 2 and Figures 4 and 5, we show the performance of all GFR estimation equations. All equations except 2 cystatin C–based equations, CKD-EPI_cys_ and 2020_Csy-B2M-BTP_ overestimated the measured GFR.

**Table 2:** Performance of CKD-EPI eGFR equations against measured GFR in study subjects

Method (*n* = 412)	Mean Bias (95 % CI) (ml/min per 1.73 m^2^)	Precision IQR (95% CI) (ml/min per 1.73 m^2^)	Accuracy				
			95% distribution of bias (ml/min per 1.73 m^2^)	RMSE (ml/min per 1.73 m^2^)	MAE (95% CI) (ml/min per 1.73 m^2^)	P_30_ (%)	P_10_(%)
CKD-EPI_Cr(2021)_	−19.2 (−21.3 to −17.0)	30.3 (25.8–34.3)	−61.5 to 23.0	28.9	22.7 (21.1–24.4)	41	11
CKD-EPI_Cr-Cys(2021)_	−6.6 (−8.4 to −4.6)	22.3 (19.5–25.7)	−44.2 to 31.0	20.3	15.6 (14.4–16.8)	55	18
CKD-EPI_Cr(2009)_	−17.0 (−19.1 to −15.0)	29.0 (25.6–33.0)	−58.9 to 24.8	27.3	21.2 (19.7–23.0)	43	14
CKD-EPI_Cr-Cys(2012)_	−0.9 (−2.7 to 1.0)	20.7 (18.0–23.1)	−36.9 to 35.2	18.4	13.8 (12.7–15.0)	60	21
CKD-EPI_Cys_	3.7 (1.73–5.5)	20.4 (17.9–23.4)	−34.2 to 41.4	19.6	14.2 (13.0–15.6)	61	23
2020_Csy-B2M-BTP_	3.4 (1.5–5.3)	22.0 (18.6–25.4)	−34.9 to 41.5	19.8	14.5 (13.3–15.9)	62	21
2020_Cr-Csy-B2M-BTP_	−4.1 (−5.8 to −2.4)	20.3 (17.6–23.4)	−38.7 to 30.5	18.2	14.0 (12.9–15.1)	59	23
EKFC_cr_	−15.0 (−16.8 to −13.0)	25.6 (22.0–28.6)	−53.7 to 23.8	24.8	19.4 (18.0–21.0)	47	16
EKFC_cys_	−0.2 (−2.0 to 1.8)	20.4 (18.2–23.6)	−39.2 to 38.8	19.9	14.4 (13.2–16.0)	60	23
EKFC_cr-cys_	−7.6 (−9.3 to −5.8)	21.1 (18.8–23.6)	−41.8 to 26.6	19.0	15.1 (14.0–16.2)	54	20

B2M, β2-microglobulin; BTP, β-trace protein; CI, confidence interval; CKD, chronic kidney disease; CKD-EPI, Chronic Kidney Disease Epidemiology Collaboration equation; Cr, creatinine; Cys, cystatin C; EKFC, European Kidney Function Consortium; eGFR, estimated glomerular filtration rate; IQR, interquartile range; MAE, mean absolute error; mGFR, measured glomerular filtration rate; P_30_, percentage of participants with eGFR within ±30% of mGFR; P_10_, percentage of participants with eGFR within ±10% of mGFR; RMSE, root mean square error.

Mean bias was expressed as the mean difference in measured GFR minus eGFR (95% bootstrapped CI).

Precision was expressed as the interquartile range (IQR) of differences in mGFR minus eGFR (95% bootstrapped CI).

95% distribution of bias was expressed as mean ± 1.96 SD.

The mean bias (95% confidence interval) was highest for CKD-EPI_cr(2021)_ (−19.2 [−21.3 to −17.0] ml/min per 1.73 m^2^) and lowest for EKFC_cys_ (−0.2 [−2.0 to 1.8] ml/min per 1.73 m^2^). Performance in terms of bias for CKD-EPI_cr(2021)_ was poorer than CKD-EPI_Cr(2009)_ (−17.0 [−19.1 to −15.0] ml/min per 1.73 m^2^) and EKFC_cr_ (−15.0 [−16.8 to −13.0] ml/min per 1.73 m^2^). All creatinine-based equations overestimated the mGFR. Inclusion of cystatin C in these equation reduced the bias as shown by CKD-EPI_Cr-Cys(2012)_ (−0.9 [−2.7 to 1.0]), CKD-EPI_Cr-Cys(2021)_ (−6.6 [−8.4 to −4.6]), EKFC_cr-cys_ (−7.6 [−9.3 to −5.8]) and 2020_Cr-Csy-B2M-BTP_ (−4.1 [−5.8 to −2.4]) whereas equation without creatinine underestimated (CKD-EPI_Cys_: 3.7 [1.73 to 5.5] and 2020_Csy-B2M-BTP_: 3.4 [1.5 to 5.3]).

Accuracy, as represented by RMSE, MAE, and P_30_/P_10_, was better for eGFR equations with cystatin C as compared to creatinine-based equations. Accuracy was similar for CKD-EPI_Cr(2021)_ (RMSE: 28.9 ml/min per 1.73 m^2^, MAE: 22.7 ml/min per 1.73 m^2^, and P_30_/P_10_: 41%/11%), CKD-EPI_Cr(2009)_ (RMSE: 27.3 ml/min per 1.73 m^2^, MAE: 21.2 ml/min per 1.73 m^2^, and P_30_/P_10_: 43%/14%), and EFKCcr (RMSE: 24.8 ml/min per 1.73 m^2^, MAE: 19.4 ml/min per 1.73 m^2^, and P_30_/P_10_: 47%/16%). As shown in Table 2, accuracy was similar for all other equations that included cystatin C, CKD-EPI_Cys_ (RMSE: 19.6 ml/min per 1.73 m^2^, MAE: 14.2 ml/min per 1.73 m^2^, and P_30_/P_10_: 61%/21%), and 2020_Csy-B2M-BTP_ (RMSE: 19.8, MAE: 14.5 and P_30_/P_10_: 62%/21%) being the better than all other equations.

Precision was better for CKD-EPI_Cys_ (20.4 [17.9–23.4] ml/min per 1.73 m^2^), EKFC_cys_ (20.4 [18.2–23.6] ml/min per 1.73 m^2^) and 2020_Cr-Csy-B2M-BTP_ (20.3 [17.6–23.4] ml/min per 1.73 m^2^) whereas it was higher for CKD-EPI_Cr(2021)_ (30.3 [25.8, 34.3] ml/min per 1.73 m^2^). As shown in Table 2 and Figure 4, 95% limit of agreement was wide for all the equations.

### Subgroup Analysis

The performance of all equations was consistent with the overall finding when data were categorized into subgroups such as potential kidney donors versus CKD and mGFR categories (<15, 15–30, >30–60, and >60 ml/min per 1.73 m^2^) (Table 3 and Table 4) as well as sex and age (<50 years and ≥50 years) (Supplementary Table S2 and Table S3). The performance of the new race-free eGFR equations (CKD-EPI_cr(2021)_ and CKD-EPI_cr-cys(2021)_) did not improve when compared to CKD-EPI equations containing creatinine or creatinine and cystatin C with race in the equations. The equation based on other endogenous markers such as cystatin C, B2M, and BTP performed similarly in potential kidney donors and CKD subjects. The performance of all these equations were similar for other subgroups (Tables 3 and 4, and Supplementary Tables S2 and S3). In sensitivity analysis for mGFR < 45 and ≥ 45 ml/min per 1.73 m^2^, performance of all the equations remained similar to performance in the overall population (Supplementary Table S4). Another analysis with respect to measured GFR was performed between the subjects with urinary inulin clearance and plasma iohexol clearance. The performance of these equations was similar; however, the bias was reduced in the group where GFR was measured with plasma clearance of iohexol (Table 5).

**Table 3:** Performance of GFR estimating equations as compared to measured GFR in potential kidney donors and subjects with CKD

Method	Mean Bias (95% CI) (ml/min per 1.73 m^2^)	Precision IQR (95% CI) (ml/min per 1.73 m^2^)	Accuracy				
			95% distribution of bias (ml/min per 1.73 m^2^)	RMSE (ml/min per 1.73 m^2^)	MAE (95% CI) (ml/min per 1.73 m^2^)	P_30_ (%)	P_10_ (%)
Potential kidney donors (*n* = 187)							
CKD-EPI_Cr(2021)_	−30.9 (−34.0 to −27.6)	32.1 (25.8– 36.0)	−74.7 to 12.9	38.1	33.1 (30.4–35.8)	38	13
CKD-EPI_Cr_Cys(2021)_	−12.4 (−15.9 to −8.9)	29.4 (24.5–35.0)	−58.7 to 33.8	26.6	22.2 (20.2–24.4)	56	20
CKD-EPI_Cr(2009)_	−28.5 (−31.8 to −25.3)	32.7 (26.0–36.7)	−72.5 to 15.5	36.3	31.2 (28.8–33.8)	40	18
CKD-EPI_Cr-Cys(2012)_	−4.1 (−7.5 to −0.7)	29.4 (24.6–35.9)	−49.9 to 41.9	23.7	19.0 (17.1–21.1)	64	24
CKD-EPI_Cys_	5.3 (1.7–8.8)	30.4 (24.5–36.4)	−42.9 to 53.6	25.1	19.2 (16.9–21.4)	68	27
2020_Csy-B2M-BTP_	8.8 (5.3–12.4)	31.1 (26.8–37.3)	−56.5 to 38.9	25.8	20.0 (17.7–22.5)	68	23
2020_Cr-Csy-B2M-BTP_	−4.6 (−7.8 to −1.2)	30.3 (26.1–34.9)	−48.7 to 39.5	22.9	18.5 (16.7–20.6)	64	27
EKFC_cr_	−23.5 (−26.4 to −20.4)	31.2 (25.9–35.8)	−67.0 to 20.0	32.3	27.4 (24.9–29.8)	47	19
EKFC_cys_	3.7 (0.4–7.1)	32.0 (24.3–35.9)	−43.4 to 50.8	24.3	18.5 (16.3–20.8)	69	27
EKFC_cr-cys_	−9.9 (−13.0 to −7.0)	29.5 (24.8–34.7)	−52.2 to 32.3	23.7	19.9 (18.1–21.6)	61	24
CKD (*n* = 225)							
CKD-EPI_Cr(2021)_	−9.5 (−11.4 to −7.6)	19.0 (13.9–21.8)	−39.2 to 20.1	17.9	14.0 (12.6–15.4)	44	10
CKD-EPI_Cr_Cys(2021)_	−1.8 (−3.3 to −0.1)	15.2 (13.0–18.0)	−26.8 to 23.3	12.9	10.1 (9.1–11.1)	56	13
CKD-EPI_Cr(2009)_	−7.5 (−9.4 to −5.6)	18.1 (13.7–21.1)	−36.4 to 21.4	16.5	12.9 (11.6–14.3)	46	12
CKD-EPI_Cr-Cys(2012)_	1.8 (0.3–3.4)	15.0 (12.7–17.3)	−22.3 to 25.9	12.4	9.5 (8.5–10.5)	56	19
CKD-EPI_Cys_	2.1 (0.4–3.7)	15.6 (13.4–18.6)	−23.9 to 28.0	13.4	10.0 (8.9–11.2)	55	20
2020_Csy-B2M-BTP_	−1.1 (−2.7 to 0.6)	15.0 (13.0–17.0)	−26.0 to 23.9	12.8	9.9 (8.8–11.0)	57	19
2020_Cr-Csy-B2M-BTP_	−3.7 (−5.3 to −2.2)	14.6 (12.2–17.0)	−27.8 to 20.3	12.8	10.2 (9.1–11.1)	54	20
EKFC_cr_	−7.9 (−9.8 to −6.1)	17.6 (13.2–20.2)	−35.4 to 19.6	16.1	12.7 (11.4–14.0)	47	14
EKFC_cys_	−3.5 (−5.6 to −1.5)	15.7 (12.3–18.5)	−32.8 to 25.9	15.3	11.1 (9.7–12.5)	52	20
EKFC_cr-cys_	−5.7 (−7.3 to −4.1)	14.3 (12.5–17.3)	−18.5 to 19.5	14.0	11.2 (10.0–12.3)	48	18

B2M, β2-microglobulin; BTP, β-trace protein; CI, confidence interval; CKD, chronic kidney disease; CKD-EPI, Chronic Kidney Disease Epidemiology Collaboration; Cr, creatinine; Cys, cystatin C; EKFC, European kidney function consortium; eGFR, Estimated glomerular filtration rate; IQR, interquartile range; MAE, mean absolute error; mGFR, measured glomerular filtration rate; P_30_, percentage of participants with eGFR within ± 30% of mGFR; P_10_, percentage of participants with eGFR within ± 10% of mGFR; RMSE, root mean square error.

Mean bias was expressed as the mean difference in mGFR minus eGFR (95% bootstrapped CI).

Precision was expressed as the interquartile range (IQR) of differences in mGFR minus eGFR (95% bootstrapped CI).

95% distribution of bias was expressed as mean ± 1.96 SD.

**Table 4:** Performance of GFR estimating equations as compared to measured GFR in participants stratified by mGFR

Method	Mean Bias (95% CI) (ml/min per 1.73 m^2^)	Precision IQR (95% CI) (ml/min per 1.73 m^2^)		Accuracy			
			95% distribution of bias (ml/min per 1.73 m^2^)	RMSE (ml/min per 1.73 m^2^)	MAE (95% CI) (ml/min per 1.73 m^2^)	P_30_ (%)	P_10_ (%)
Study participants with mGFR >60 ml/min per 1.73 m^2^ (*n* = 164)							
CKD-EPI_Cr(2021)_	−24.3 (−27.9 to −21.0)	30.6 (26.6–37.3)	−67.3 to 18.6	32.7	28.2 (25.5–30.7)	48.8	16
CKD-EPI_Cr_Cys(2021)_	−6.8 (−10.5 to −3.1)	36.1 (28.1–43.1)	−54.7 to 41.1	25.3	21.1 (19.0–23.0)	62.8	21
CKD-EPI_Cr(2009)_	−22.2 (−25.8 to −18.8)	31.8 (26.4–38.2)	−66.0 to 21.5	31.5	26.9 (24.3–29.3)	49.4	20
CKD-EPI_Cr-Cys(2012)_	1.4 (−2.1 to 5.0)	35.4 (28.1–40.4)	−45.6 to 48.3	23.9	19.1 (17.2–21.3)	67.1	27
CKD-EPI_Cys_	10.6 (6.9–14.6)	33.4 (29.1–41.4)	−38.3 to 59.4	27.0	21.1 (18.8–23.9)	67.7	24
2020_Csy-B2M-BTP_	14.8 (11.1–18.4)	30.5 (23.7–35.3)	−60.6 to 31.0	27.6	21.9 (19.4–24.6)	68.9	21
2020_Cr-Csy-B2M-BTP_	1.3 (−2.1 to 4.6)	31.3 (25.8–37.6)	−44.3 to 41.7	21.9	17.5 (15.5–19.5)	72	30
EKFC_cr_	−17.4 (−20.7 to −14.0)	30.1 (24.6–35.2)	−59.1 to 24.4	27.4	23.1 (20.7–25.3)	58	23
EKFC_cys_	8.9 (5.1–12.7)	31.4 (26.7–38.8)	−38.2 to 55.9	25.5	19.7 (17.4–22.3)	70	26
EKFC_cr-cys_	−4.2 (−7.2 to −1.1)	30.7 (25.9–36.1)	−45.5 to 37.0	21.4	18.0 (16.2–19.6)	72	25
Study participants with mGFR >30–60 ml/min per 1.73 m^2^ (*n* = 131)							
CKD-EPI_Cr(2021)_	−19.5 (−23.8 to −15.3)	39.4 (20.0–47.6)	−68.7 to 29.8	31.7	23.3 (19.9–26.9)	47	12
CKD-EPI_Cr_Cys(2021)_	−6.5 (−9.8 to −3.5)	24.2 (18.7–30.9)	−42.8 to 29.8	19.6	14.8 (12.6–17.0)	57	19
CKD-EPI_Cr(2009)_	−16.7 (−20.9 to −12.6)	38.7 (20.9–45.9)	−64.8 to 31.4	29.6	21.4 (18.1–24.9)	49	15
CKD-EPI_Cr-Cys(2012)_	−1.3 (−4.2 to 1.3)	22.0 (16.8–27.2)	−33.9 to 31.3	16.6	13.1 (11.3–14.8)	60	20
CKD-EPI_Cys_	1.9 (−0.7 to 4.1)	18.1 (15.1–23.1)	−27.0 to 30.8	14.8	11.7 (10.2–13.3)	60	25
2020_Csy-B2M-BTP_	−0.7 (−2.8 to 1.2)	15.6 (12.6–19.0)	−25.0 to 23.6	12.4	9.8 (8.4–11.0)	73	27
2020_Cr-Csy-B2M-BTP_	−6.7 (−9.3 to −4.2)	19.3 (15.8–24.6)	−36.9 to 23.5	16.8	12.7 (10.7–14.5)	63	25
EKFC_cr_	−15.7 (−19.9 to −11.8)	33.8 (20.7–40.8)	−60.3 to 28.9	27.6	20.2 (17.1–23.4)	49	18
EKFC_cys_	−2.7 (−5.3 to −0.3)	18.0 (14.3–21.5)	−29.3 to 24.0	13.8	10.8 (9.1–12.3)	69	25
EKFC_cr-cys_	−9.2 (−12.2 to −6.3)	24.0 (17.7–28.2)	−42.5 to 24.2	19.3	14.5 (12.4–16.7)	54	22
Study participants with mGFR 15–30 ml/min per 1.73 m^2^ (*n* = 97)							
CKD-EPI_Cr(2021)_	−11.9 (−15.0 to −9.1)	16.8 (12.8–23.7)	−39.5 to 15.6	18.4	14.9 (12.8–17.1)	29	4
CKD-EPI_Cr_Cys(2021)_	−6.1 (−8.2 to −4.1)	12.6 (9.1–16.6)	−25.2 to 13.1	11.5	9.0 (7.7–10.4)	51	12
CKD-EPI_Cr(2009)_	−10.3 (−13.2 to −7.4)	16.8 (13.0–23.0)	−36.7 to 16.2	16.9	13.7 (11.9–15.6)	31	5
CKD-EPI_Cr-Cys(2012)_	−3.1 (−5.0 to −1.3)	11.6 (8.7–15.1)	−20.7 to 14.6	9.5	7.5 (6.4–8.6)	55	16
CKD-EPI_Cys_	−3.9 (−5.8 to −2.1)	11.8 (8.8–14.9)	−20.7 to 12.9	9.4	7.2 (6.1–8.5)	59	21
2020_Csy-B2M-BTP_	−7.5 (−9.4 to −5.6)	9.7 (6.6–15.3)	−25.1 to 10.1	11.6	9.1 (7.7–10.45)	46	17
2020_Cr-Csy-B2M-BTP_	−8.6 (−10.8 to −6.6)	11.4 (8.8–16.2)	−28.2 to 11.0	13.1	10.4 (8.8–11.9)	41	13
EKFC_cr_	−10.9 (−13.4 to −8.4)	15.1 (12.2–22.3)	−35.1 to 14.2	16.8	13.7 (11.8–15.7)	32	5
EKFC_cys_	−9.0 (−10.7 to −7.3)	12.5 (8.7–15.0)	−26.0 to 8.0	12.5	9.8 (8.3–11.3)	40	18
EKFC_cr-cys_	−10.0 (−11.7 to −8.0)	12.6 (9.1–16.6)	−29.0 to 9.1	13.9	11.3 (9.8–12.9)	33	14
Study participants with mGFR <15 ml/min per 1.73 m^2^ (*n* = 20)							
CKD-EPI_Cr(2021)_	−11.1 (−14.0 to −8.6)	6.5 (3.3–10.6)	−23.4 to 1.3	12.7	11.1 (8.6–13.8)	5	5
CKD-EPI_Cr_Cys(2021)_	−7.7 (−9.8 to −6.0)	4.5 (2.5–8.0)	−16.3 to 1.0	8.8	7.7 (5.8–9.6)	15	10
CKD-EPI_Cr(2009)_	−9.9 (−12.6 to −7.5)	5.9 (3.3–9.7)	−21.5 to 1.8	11.4	9.9 (7.6–12.4)	10	5
CKD-EPI_Cr-Cys(2012)_	−5.6 (−7.5 to −4.1)	3.5 (2.0–7.0)	−13.3 to 2.0	6.8	5.7 (4.2–7.4)	30	10
CKD-EPI_Cys_	−7.0 (−9.1 to −5.2)	6.1 (3.7–10.0)	−15.7 to 1.6	8.3	7.1 (5.2–9.1)	25	10
2020_Csy-B2M-BTP_	−10.8 (−12.7 to −9.0)	4.7 (2.4–9.7)	−18.9 to −2.6	11.5	10.7 (9.0–12.5)	5	0
2020_Cr-Csy-B2M-BTP_	−10.7 (−12.7 to −8.8)	6.2 (3.2–9.4)	−19.5 to −1.9	11.5	10.7 (8.7–12.5)	5	0
EKFC_cr_	−10.7 (−13.1 to −8.3)	5.2 (3.5–9.3)	−22.0 to 0.7	12.0	10.7 (8.3–13.2)	10	5
EKFC_cys_	−17.0 (−28.0 to −10.4)	8.0 (3.7–13.0)	−61.5 to 27.5	27.9	17.0 (10.3–27.4)	5	0
EKFC_cr-cys_	−13.8 (−19.5 to −10.0)	4.7 (2.5–13.0)	−37.0 to 9.3	18.0	13.8 (9.7–19.1)	5	0

B2M, β2-microglobulin; BTP, β-trace protein; CI, confidence interval; CKD, chronic kidney disease; CKD-EPI, Chronic Kidney Disease Epidemiology Collaboration; Cr, creatinine; Cys, cystatin C; EKFC, European Kidney Function Consortium; eGFR, estimated glomerular filtration rate; IQR, interquartile range; MAE, mean absolute error; mGFR, measured glomerular filtration rate; P_30_, percentage of participants with eGFR within ±30% of mGFR; P_10_, percentage of participants with eGFR within ±10% of mGFR; RMSE, root mean square error.

Mean bias was expressed as the mean difference in mGFR minus eGFR (95% bootstrapped CI).

Precision was expressed as the interquartile range (IQR) of differences in mGFR minus eGFR (95% bootstrapped CI).

95% distribution of bias was expressed as mean ± 1.96 SD.

**Table 5:** Performance of GFR estimating equations as compared to measured GFR using urinary inulin clearance and plasma Iohexol clearance

Method	Mean Bias (95% CI) (ml/min per 1.73 m^2^)	Precision IQR (95% CI) (ml/min per 1.73 m^2^)	Accuracy				
			95% distribution of bias (ml/min per 1.73 m^2^)	RMSE (ml/min per 1.73 m^2^)	MAE (95% CI) (ml/min per 1.73 m^2^)	P_30_ (%)	P_10_ (%)
Inulin (*n* = 130)							
CKD-EPI_Cr(2021)_	−26.0 (−28.8 to −23.4)	27.5 (22.3 to 30.3)	−58.7 to −9.4	30.9	26.5 (23.8–29.3)	21	3
CKD-EPI_Cr-Cys(2021)_	−13.7 (−16.3 to −11.3)	17.1 (11.9–21.7)	−42.2 to 14.9	20.0	15.1 (12.9–17.4)	53	17
CKD-EPI_Cr(2009)_	−24.2 (−27.1 to −21.5)	26.6 (21.0–29.3)	−56.7 to 8.2	29.3	24.8 (22.1–27.6)	23	5
CKD-EPI_Cr-Cys(2012)_	−7.7 (−10.0 to −5.4)	13.1 (10.0–16.5)	−21.1 to 18.7	15.4	11.0 (9.1–12.8)	69	27
CKD-EPI_Cys_	−3.2 (−5.6 to −0.9)	13.3 (10.0–16.2)	−30.1 to 23.8	14.1	10.0 (8.5–11.8)	74	27
2020_Csy-B2M-BTP_	0.8 (−1.8 to 3.40	16.1 (11.7–21.8)	−28.4 to 30.1	14.9	11.2 (9.6–12.9)	67	25
2020_Cr-Csy-B2M-BTP_	−9.6 (−11.7 to −7.6)	12.5 (10.2–16.5)	−33.3 to 14.2	15.4	12.3 (10.8–14.0)	55	23
EKFC_cr_	−21.5 (−24.1 to −19.0)	18.5 (16.1–23.4)	−50.0 to 7.1	25.9	22.1 (19.7–24.5)	29	8
EKFC_cys_	−6.8 (−9.7 to −4.1)	12.9 (9.9–15.5)	−38.1 to 24.5	17.3	11.7 (9.7–14.3)	63	28
EKFC_cr-cys_	−14.1 (−16.4 to −11.6)	12.6 (10.6–15.4)	−39.5 to 11.3	19.1	15.7 (13.8–17.8)	45	15
Iohexol (*n* = 282)							
CKD-EPI_Cr(2021)_	−16.1 (−18.9 to −13.4)	30.8 (25.3–38.0)	−60.8 to 28.6	27.8	20.9 (18.9–23.1)	50.7	15
CKD-EPI_Cr-Cys(2021)_	−3.3 (−5.6 to −0.9)	25.6 (21.0–29.8)	−42.8 to 36.2	20.4	15.8 (14.2–17.4)	56.4	18
CKD-EPI_Cr(2009)_	−13.7 (−16.5 to −11.1)	30.1 (24.5–36.7)	−57.8 to 30.4	26.3	19.6 (17.6–21.6)	52	18
CKD-EPI_Cr-Cys(2012)_	−2.3 (−0.02 to 4.6)	24.8 (19.8–28.5)	−40.5 to 36.0	19.6	15.1 (13.6–16.6)	56	18
CKD-EPI_Cys_	6.7 (4.4–9.3)	25.8 (21.7–29.6)	−33.8 to 47.2	21.7	16.1 (14.5–17.8)	55	21
2020_Csy-B2M-BTP_	4.6 (2.3–7.0)	24.9 (20.9–29.1)	−37.0 to 46.2	21.7	16.0 (14.3–17.7)	60	19
2020_Cr-Csy-B2M-BTP_	−1.6 (−3.8 to 0.7)	23.5 (19.9–27.3)	−39.2 to 36.0	19.2	14.7 (13.2–16.2)	60	23
EKFC_cr_	−12.0 (−14.4 to −9.4)	27.2 (21.9–32.7)	−53.4 to 29.4	24.3	18.1 (16.3–20.0)	55	20
EKFC_cys_	2.8 (0.3–5.2)	24.3 (20.9–29.9)	−38.0 to 43.6	21.0	15.7 (14.1–17.2)	58	21
EKFC_cr-cys_	−4.6 (−6.8 to −2.4)	24.5 (20.0–28.3)	−40.7 to 31.6	19.0	14.9 (13.4–16.2)	57	23

B2M, β2-microglobulin; BTP, β-trace protein; CI, confidence interval; CKD, chronic kidney disease; CKD-EPI, Chronic Kidney Disease Epidemiology Collaboration; Cr, creatinine; Cys, cystatin C; EKFC, European Kidney Function Consortium; eGFR, estimated glomerular filtration rate; IQR, interquartile range; MAE, mean absolute error; mGFR, measured glomerular filtration rate; P_30_, percentage of participants with eGFR within ±30% of mGFR; P_10_, percentage of participants with eGFR within ±10% of mGFR; RMSE, root mean square error.

Mean bias was expressed as the mean difference in mGFR minus eGFR (95% bootstrapped CI).

Precision was expressed as the interquartile range (IQR) of differences in mGFR minus eGFR (95% bootstrapped CI).

95% distribution of bias was expressed as mean ± 1.96 SD.

## Discussion

This study shows that all GFR estimation equations, including the new race-free equations, perform poorly in the north Indian Population. The 2012 CKD-EPI_cys_ was slightly better than creatinine-based equations but could achieve a P_30_ of only 61%, which is well-short of the 80% acceptability threshold. Although the cystatin C–based equations reduced or even eliminated the eGFR overestimation, they did not improve performance in terms of bias and P_30_, pointing to the need to reexpress the GFR estimation equation in the Indian population. Additional markers, as in the 2020_Csy-B2M-BTP_ and 2020_Cr-Csy-B2M-BTP_ equations, did not substantially improve the performance over the CKD-EPI_Cys_ equation. Overall, the eGFR equations utilizing markers other than serum creatinine slightly underestimated the mGFR, and the inclusion of creatinine to the equations led to GFR overestimation.

Poor performance of the eGFR equations in the Indian population may lead to inappropriate diagnosis (chiefly underdiagnosis using the creatinine-based equations), misclassification, and staging of CKD. Misclassifications can lead to changes in estimates of CKD prevalence in the population. However, the routine use of eGFR in clinical practice conflicts the average population value with the individual value. Fortunately, for most practical purposes, the specific value of GFR often does not change management. Exceptions where the knowledge of GFR is essential include the determination of the suitability of kidney donors, deciding on drug dosing, withholding drugs such as sodium-glucose cotransporter-2 inhibitors or mineralocorticoids at low GFR or when recruiting subjects for clinical trials.

The poor performance of creatinine-based eGFR equations in our population could be due to non-GFR determinants such as comparatively lower muscle mass, small body structure, and vegetarian dietary habits. As was seen in this study, meat consumption in India is substantially lower than that of the US population. Meat consumption can impact eGFR formulae that use serum creatinine and can explain some of the discrepancies. The eGFR equations based on other endogenous markers, such as cystatin C, BTP, and B2M, are less affected than creatinine by non-GFR determinants. Although the serum levels of these markers have been strongly correlated with measured GFR and less strongly correlated with age, sex, and race in comparison to serum creatinine,^21^ equations including these endogenous markers also performed poorly, suggesting that the levels of these markers are also affected by yet undefined non-GFR determinants.

A number of studies^5^^,^^20^^,^^22^ have reported recently on race-free CKD-EPI equations. The new CKD-EPI_cr(2021)_ equation increased the population estimate of CKD prevalence in the Black population compared with the CKD-EPI_cr(2009)_ equation in USA.^5^ The comparison of CKD-EPI_cr(2009)_ equation and CKD-EPI_cr(2021)_ among US living kidney donors from the Scientific Registry of Transplant Recipients reported decreased GFR in Blacks and increased GFR in non-Blacks with the use of the latter.^23^ In the non-Black Danish population, CKD prevalence (4.2%) decreased with CKD-EPI_cr(2021)_ equations than the original equation (5.5%).^24^ However, the performance of these equations in the Asian populations has been uniformly poor. A study from Korea reported greater bias with CKD-EPI_cr(2021)_ eGFR equation as compared to CKD-EPI_cr(2009)_ equation, leading to a change in proportion of CKD stage 3 from 3.4% for CKD-EPI_cr(2009)_ to 2.6% for CKD-EPI_cr(2021)_, and CKD prevalence in the general population from 11% for CKD-EPI_cr(2009)_ to 10.2% for CKD-EPI_cr(2021)_.^25^ Similarly, a recent study from Pakistan found no improvement in precision and accuracy for the new race-free equation because it increased the overestimation against mGFR compared to CKD-EPI_cr(2009)_ equation (median bias −8.94 ml/min per 1.73 m^2^ for CKD-EPI_cr(20__21__)_ and −6.76 ml/min per 1.73 m^2^ for CKD-EPI_cr(20__09__)_).^26^ In another recent study from India, CKD-EPI_cr(2021)_ equation increased the eGFR, lowering the estimate of kidney disease burden.^27^ We have used 2 different gold standard methods for measuring GFR. Both do not give identical results, with plasma clearance being slightly higher than urinary clearance due to nonrenal clearance. The difference in the performance of these equations may also be due to the methods of GFR measurement, which use different exogenous markers such as iohexol, iothalamate, and ethylenediaminetetraacetic acid.

The inclusion of cystatin C also has the potential to eliminate the necessity for adjustments based on race.^28^ The eGFR equation with cystatin C developed by the European Kidney Function Consortium (EKFC eGFRcyst) was reported to have a better accuracy than CKD-EPI_cr(2021)_ in cohorts from Europe, the USA, and Africa.^20^ In a recent meta-analysis of 35 studies with 23,667 participants, CKD-EPI_cys_ had the least bias and provided more accurate estimates of mGFR than CKD-EPI_cr-cys(2012)_ and CKD-EPI_cr(2009)_.^29^ Notable was the poor performance of CKD-EPI_cys_ eGFR equation in the South Asian population, showing underestimation by a mean bias of 12.7 ml/min per 1.73 m^2^ against mGFR,^30^ which is similar to the current study.

B2M and BTP are low-molecular-weight proteins, freely filtered by the glomerulus with no tubular secretion or extrarenal elimination and concentrations (plasma and urinary) of these proteins can increase early after a kidney insult.^31^, ^32^, ^33^ They are less dependent on age, sex, and race and have been proposed as alternate filtration markers for inclusion in a panel of eGFR estimating equations. In our study, we observed underestimation with 3-marker (2020_Csy-B2M-BTP_) and overestimation with 4-marker (2020Cr-Csy-B2M-BTP), although the P_30_ and RMSE were similar for both equations. Our findings are similar to those reported from Pakistan, where the 2020_Csy-B2M-BTP_ (P_30_: 70.7%) and 4–2020_Cr-Csy-B2M-BTP_ (P_30_: 81.3%) did not perform better than the CKD-EPI_cr_−PK (P_30_: 82.4%) equation.^26^ In contrast, a study from China showed that equations using a panel of endogenous filtration markers, including B2M and BTP in addition to creatinine and cystatin C were more accurate (1–P_30_: 10.4%; RMSE of 21.4) than CKD-EPI_cr-cys(2012)_ (1−P_30_: 13.8%; RMSE of 23.2).^34^ However, the improvements in accuracy were not clinically significant because they did not change the classification or reclassification of mGFR categories in this cohort.

The strength of this study is the use of rigorous standardized measurement techniques. However, the sample size is relatively small, and represents people drawn from northern India. We did not collect information on lung disease, edema, ascites, or smoking status, which can influence GFR. In addition, the study did not include bladder scan to ensure bladder emptying; and sampling time for iohexol plasma clearance was limited to 4 hours. Finally, we did not analyze the reclassification index.

In conclusion, the new race-free CKD-EPI equations did not enhance the accuracy or reduce the bias against measured GFR compared to older CKD-EPI equations in Indians. The study demonstrates the fallacy of estimating GFR and enforcing calculated parameters, and supports the need to develop new eGFR equations that include a representative, diverse Indian population with a wide range of GFR. Finally, given the caveats with eGFR, standardizing GFR measurement is vital so that nephrologists are able to optimize patient care.

## Disclosure

VJ has received grant funding from GSK, Baxter Healthcare, and Biocon; and honoraria from Bayer, AstraZeneca, Boehringer Ingelheim, NephroPlus, and Zydus Cadilla under the policy of all honoraria being paid to the organization. All the other authors declared no competing interests.

## Supplementary Material

Supplementary File (PDF) **Table S1.** Estimating GFR equations. **Table S2.** Performance of GFR estimating equations as compared to measured GFR in males and females. **Table S3.** Performance of GFR estimating equations as compared to measured GFR in age subgroup. **Table S4.** Performance of GFR estimating equations as compared to measured GFR in participants with mGFR: < 45 and ≥45 ml/min per 1.73 m^2^.

## Footnotes

Supplementary File (PDF)
**Table S1.** Estimating GFR equations. **Table S2.** Performance of GFR estimating equations as compared to measured GFR in males and females. **Table S3.** Performance of GFR estimating equations as compared to measured GFR in age subgroup. **Table S4.** Performance of GFR estimating equations as compared to measured GFR in participants with mGFR: < 45 and ≥45 ml/min per 1.73 m^2^.
