# Supplementary material for: Evaluation of Race-Neutral Glomerular Filtration Rate Estimating Equations in an Indian Population
Source: Kidney Int Rep. 2024 Oct 10;9(12):3414–26. doi: 10.1016/j.ekir.2024.09.020 (PMC11652306; doi:10.1016/j.ekir.2024.09.020)
Supplement: Supplementary File (PDF) — Table S1. Estimating GFR equations. Table S2. Performance of GFR estimating equations as compared to measured GFR in males and females. Table S3. Performance of GFR estimating equations as compared to measured GFR in age subgroup. Table S4. Performance of GFR estimating equations as compared to measured GFR in participants with mGFR: < 45 and ≥45 ml/min per 1.73 m2. [file mmc1.pdf]

Supplementary files

Evaluation of Race-Neutral Glomerular Filtration Rate Estimating Equations in an Indian Population

Ashok Kumar Yadav[1], Jaskiran Kaur[1], Prabhjot Kaur[2], Kajal Kamboj[2], Yoshinari Yasuda[3,4], Masaru Horio[5], Arnab Pal[6], Nusrat Shafiq[7], Nancy Sahni[8], Harbir Singh Kohli[2], Seiichi Matsuo[3], Vivek Kumar[2], Vivekanand Jha[9,10,11]

[1]Department of Experimental Medicine and Biology, Postgraduate Institute of Medical Education and Research, Chandigarh, [2]Department of Nephrology, Postgraduate Institute of Medical Education and Research, Chandigarh, [3]Department of Nephrology, Nagoya University Graduate School of Medicine, Nagoya, Japan. [4]Department of CKD Initiatives, Nagoya University Graduate School of Medicine, Nagoya, Japan. [5]Division of Health Sciences, Graduate School of Medicine, Osaka University, Osaka, Japan. [6]Department of Biochemistry, Postgraduate Institute of Medical Education and Research, Chandigarh, [7]Department of Pharmacology, Postgraduate Institute of Medical Education and Research, Chandigarh, [8]Department of Dietetics, Postgraduate Institute of Medical Education and Research, Chandigarh, [9]The George Institute for Global Health, New Delhi, [10]School of Public Health, Imperial College, London, UK, [11]Manipal Academy of Higher Education, Manipal, India

SUPPLEMENTARY TABLES

Table S1. Estimating GFR equations

eGFR equation (ml/min/1.73m^2)	Formula		
CKD-EPI$_{Cr(2021)}$	142 x min (SCr/κ, 1)$^{\alpha}$ x max (SCr /κ, 1)$^{-1.200}$ x 0.9938Age x 1.012 [if female]		
CKD-EPI$_{Cr\text{-}Cys(2021)}$	135 x min (SCr/κ, 1)$^{\alpha}$ x max (SCr /κ, 1)$^{-0.544}$ x min (Scys/0.8, 1)$^{-0.323}$ x max (Scys/0.8, 1)$^{-0.778}$ x 0.9961Age x 0.963 [if female]		
CKD-EPI$_{Cr(2009)}$	141 x min (SCr/κ, 1)$^{\alpha}$ x max (SCr /κ, 1)$^{-1.209}$ x 0.993Age x 1.018 (if female)		
CKD-EPI$_{Cr\text{-}Cys(2012)}$	135 x min (SCr/κ, 1)$^{\alpha}$ x max (SCr /κ, 1)$^{-0.601}$ x min (Scys/0.8, 1)$^{-0.375}$ x max (Scys/0.8, 1)$^{-0.711}$ x 0.9952Age x 0.969 [if female]		
CKD-EPI$_{Cys}$	133 x min (Scys/0.8, 1)$^{-0.499}$ x max (Scys/0.8, 1)$^{-1.328}$ x 0.996Age x 0.932 (if female)		
2020 Cystatin C–B2M-BTP (2020$_{Csy\text{-}B2M\text{-}BTP}$)	120 × min(Scys/0.8,1)$^{−0.876}$ × max(Scys/0.8,1)$^{−0.697}$ × B2M$^{−0.205}$ × min(SBTP/0.6,1)$^{0.038}$ × max(SBTP/0.6,1)$^{−0.243}$ × 0.999^{age} [×0.922 if female].		
2020 Creatinine–Cystatin C–B2M-BTP (2020$_{Cr\text{-}Csy\text{-}B2M\text{-}BTP}$)	131 × min(Scr/k,1)$^{\alpha}$ × max(Scr/k,1$^{)−0.471}$ × min(Scys/0.8,1)$^{−0.519}$ × max(Scys/0.8,1)$^{−0.423}$ × SB2M$^{−0.103}$ × min(SBTP/0.6,1)$^{−0.004}$ × max(SBTP/0.6,1)$^{−0.177}$ × 0.996^{age} [× 0.937 if female]		
EKFC$_{cr}$	If age= 2–40	Scr/Q<1	107.3×(Scr/*Q*)$^{−0.322}$
		Scr/Q ≥1	107.3× (Scr/*Q*)$^{−1.132}$
	If age >40	Scr/Q<1	107.3× (Scr/*Q*)$^{−0.322}$×0.990$^{(Age\text{-}40)}$
		Scr/Q ≥1	107.3× (Scr/*Q*)$^{−1.132}$×0.990$^{(Age\text{-}40)}$

$EKFC_{cys}$	If age 2-40	ScysC/Q < 1.0	$107.3 \text{ x } (SCysC/Q)^{-0.322}$
		ScysC/Q ≥ 1.0	$107.3 \text{ x } (SCysC/Q)^{-1.132}$
	>40	ScysC/Q < 1.0	$107.3 \text{ x } (SCysC/Q)^{-0.322} \text{ x } 0.990^{(Age-40)}$
		ScysC/Q ≥ 1.0	$107.3 \text{ x } (SCysC/Q)^{-1.132} \text{ x } 0.990^{(Age-40)}$
	>50	ScysC/Q' < 1.0<br>Q' = Q+0.005x(Age – 50)	$107.3 \text{ x } (SCysC/Q')^{-0.322} \text{ x } 0.990^{(Age-40)}$
		ScysC/Q' ≥ 1.0<br>Q' = Q+0.005x(Age – 50)	$107.3 \text{ x } (SCysC/Q')^{-1.132} \text{ x } 0.990^{(Age-40)}$
$EKFC_{cr\text{-}cys}$	$(eGFR(EKFC_{Cr}) + eGFR(EKFC_{Cys})) / 2$		

κ = 0.7 (females) or 0.9 (males),

For CKD-$EPI_{cr(2021)}$ α = -0.241 (females) or -0.302 (males), For CKD-$EPI_{cr\text{-}cys(2021)}$ α = -0.219 (females) or -0.144 (males), For CKD-$EPI_{cr(2009)}$ α = -0.329 (females) or -0.411 (males), For CKD-$EPI_{cr\text{-}cys(2012)}$ α = -0.248 (females) or -0.207 (males), For 2020 Creatinine–Cystatin C–B2M-BTP α = −0.243 for women and −0.295 for men.

CKD-EPI: Chronic Kidney Disease Epidemiology Collaboration, Cr: Creatinine, Cys: cystatin C, EKFC: European kidney function consortium, eGFR: Estimated glomerular filtration rate, max: Maximum, min: Minimum, Scr: serum creatinine, B2M; beta 2 macroglobulin, BTP: beta trace protein,

Q value calculations for ages 2–25 years:

Males: $\ln(Q) = 3.200 + 0.259 \times Age - 0.543 \times \ln_{(Age)} - 0.00763 \times Age^2 + 0.0000790 \times Age^3$.

Females: $\ln(Q) = 3.080 + 0.177 \times Age - 0.223 \times \ln_{(Age)} - 0.00596 \times Age^2 + 0.0000686 \times Age^3$.

Q value calculations for ages >25 years:

Males: *Q* = 80 µmol/L (0.90 mg/dL).

Females: *Q* = 62 µmol/L (0.70 mg/dL).

Scr and *Q* are reported in µmol/L (to convert to mg/dL, divide by 88.4).

Table S2. Performance of GFR estimating equations as compared to measured GFR in male and female

Method	Mean bias (95%CI) (ml/min/1.73m²)	Precision IQR (95% CI) (ml/min/1.73m²)	Accuracy				
			95% distribution of bias (ml/min/1.73m²)	RMSE (ml/min/1.73m²)	MAE (95% CI) (ml/min/1.73m²)	P_{30} (%)	P_{10} (%)
**Males (n=208)**							
CKD-EPI$_{Cr(2021)}$	-14.7 (-17.8, -11.8)	25.3 (19.3, 32.2)	-54.9 to 25.6	25.2	19.7 (17.6, 21.9)	41.3	9.1
CKD-EPI$_{Cr_Cys(2021)}$	-3.8 (-5.9, -1.6)	18.0 (14.8, 22.2)	-35.3 to 27.7	16.5	12.9 (11.5, 14.3)	54.8	18.8
CKD-EPI$_{Cr(2009)}$	-12.2 (-15.2, -9.5)	24.2 (19.4, 30.4)	-51.4 to 27.0	23.4	18.2 (16.2, 20.2)	41.8	12.5
CKD-EPI$_{Cr-Cys(2012)}$	1.3 (-0.8 , 3.4)	16.8 (14.2, 21.3)	-28.6 to 31.1	15.3	11.5 (10.2, 12.9)	57.2	22.1
CKD-EPI$_{Cys}$	3.4 (1.4 , 5.5)	17.9 (14.9, 21.5)	-27.0 to 33.9	16.0	11.7 (10.2, 13.2)	62.5	21.6
2020$_{Csy-B2M-BTP}$	2.8 (0.6 , 5.1)	17.6 (15.3, 21.5)	-28.7 to 34.3	16.3	12.0 (10.5, 13.6)	61.1	22.1
2020$_{Cr-Csy-B2M-BTP}$	-4.3 (-6.5, -2.2)	17.0 (14.3, 21.0)	-34.5 to 25.9	16.0	12.4 (11.2, 13.8)	56.3	22.1
EKFC$_{cr}$	-11.4 (-14.2, -8.7)	22.2 (17.7, 28.4)	-48.2 to 25.5	21.9	17.2 (15.3, 19.0)	45.7	15.4
EKFC$_{cys}$	-1.8 (-4.1, 0.6)	17.6 (14.5, 22.0)	-35.6 to 32.0	17.3	12.2 (10.7, 14.0)	60.1	25.0
EKFC$_{cr-cys}$	-6.6 (-8.6 ,-4.4)	19.9 (15.5, 23.3)	-37.5 to 24.4	17.1	13.7 (12.3, 15.0)	51.4	16.3
**Females (n=204)**							
CKD-EPI$_{Cr(2021)}$	-23.9 (-26.8 , -20.9)	32.3 (28.1, 37.9)	-66.3 to 18.5	32.2	25.8 (23.2, 28.4)	41.7	13.7
CKD-EPI$_{Cr_Cys(2021)}$	-9.4 (-12.5, -6.2)	27.3 (23.0, 32.3)	-51.7 to 32.9	23.5	18.4 (16.5, 20.5)	56.9	16.7
CKD-EPI$_{Cr(2009)}$	-22.0 (-25.0 , -19.0)	31.8 (27.1, 37.6)	-64.4 to 20.4	30.8	24.3 (21.8, 27.0)	44.6	16.2
CKD-EPI$_{Cr-Cys(2012)}$	-3.0 (-6.0, -0.01)	25.6 (20.9, 29.5)	-44.1 to 38.1	21.1	16.2 (14.3, 18.0)	62.7	20.1
CKD-EPI$_{Cys}$	3.7 (0.6, 7.0)	24.5 (19.3, 30.0)	-40.4 to 47.8	22.6	16.9 (14.7, 19.0)	59.3	24.0
2020$_{Csy-B2M-BTP}$	4.5 (1.1, 7.4)	28.2 (23.2, 31.8)	-39.6 to 48.6	22.3	17.0 (15.0, 19.1)	62.71	19.1

$2020_{Cr-Csy-B2M-BTP}$	-3.9 (-6.8,1.0)	25.3 (20.0, 28.7)	-34.8 to 42.6	20.1	15.5 (13.9, 17.3)	60.8	23.5
$EKFC_{cr}$	-18.7 (-21.4,-15.8)	27.4 (22.7, 33.0)	-58.1 to 20.8	27.4	21.6 (19.3, 23.9)	48.0	17.2
$EKFC_{cys}$	1.4 (-1.7, 4.6)	23.4 (19.0, 31.0)	-42.2 to 45.0	22.2	16.6 (14.7, 18.7)	58.8	21.1
$EKFC_{cr-cys}$	-8.7 (-11.4, -5.9)	23.5 (20.2, 27.7)	-45.8 to 28.5	20.8	16.6 (15.0, 18.3)	55.9	24.5

B2M: β2-Microglobulin, BTP: β-Trace Protein, CKD: Chronic Kidney Disease, CKD-EPI: Chronic Kidney Disease Epidemiology Collaboration, Cr: Creatinine, Cys: cystatin C, EKFC: European kidney function consortium, eGFR: Estimated glomerular filtration rate, mGFR: Measured glomerular filtration rate, MAE: mean absolute error, P_{30}: Percentage of participants with eGFR within ±30% of mGFR, P_{10}: Percentage of participants with eGFR within ±10% of mGFR, RMSE: root mean square error.
Bias was expressed as the mean difference in measured GFR minus estimated GFR (95% bootstrapped confidence interval).
Precision was expressed as the interquartile range (IQR) of differences in measured GFR minus estimated GFR (95% bootstrapped confidence interval).
95% distribution of bias was expressed as mean ± 1.96*Standard deviation.

Table S3. Performance of GFR estimating equations as compared to measured GFR in age subgroup

Method	Mean bias (95%CI) (ml/min/1.73m^2)	Precision IQR (95% CI) (ml/min/1.73m^2)	Accuracy				
			95% distribution of bias (ml/min/1.73m^2)	RMSE (ml/min/1.73m^2)	MAE (95% CI) (ml/min/1.73m^2)	P_{30} (%)	P_{10}(%)
**Age ≥ 50 years (n=189)**							
CKD-EPI$_{Cr(2021)}$	-17.6 (-20.8, -14.8)	28.0 (22.9, 34.3)	-57.8 to 22.6	27.0	21.5 (19.1, 23.8)	38.6	10.6
CKD-EPI$_{Cr_Cys(2021)}$	-4.3 (-6.7, -1.8)	22.1 (17.0, 27.0)	-38.0 to 29.5	17.7	14.2 (12.7, 5.7)	53.4	16.4
CKD-EPI$_{Cr(2009)}$	-14.7 (-17.8,-11.9)	27.8 (21.8, 33.2)	-53.6 to 24.2	24.6	19.4 (17.3, 21.6)	41.8	14.8
CKD-EPI$_{Cr-Cys(2012)}$	1.4 (-1.0, 3.7)	19.8 (15.9, 25.3)	-30.9 to 33.6	16.5	12.7 (11.1, 14.2)	59.6	21.2
CKD-EPI$_{Cys}$	5.7 (3.0, 8.3)	20.9 (17.2, 25.9)	-29.8 to 41.2	18.9	13.7 (11.9, 15.6)	59.2	21.7
2020$_{Csy-B2M-BTP}$	3.0 (0.3, 5.7)	20.7 (16.7, 26.7)	-32.6 to 38.7	18.4	13.8 (12.1, 15.6)	60.8	22.8
2020$_{Cr-Csy-B2M-BTP}$	-3.3 (-5.8,-0.8)	21.3 (16.1, 26.0)	-36.1 to 29.6	17.0	13.5 (12.0, 15.0)	54.5	22.8
EKFC$_{cr}$	-12.5 (-15.3, -9.9)	26.9 (19.7, 31.9)	-49.0 to 24.0	22.4	17.9 (16.0, 19.8)	45.0	15.3
EKFC$_{cys}$	2.3 (-0.4, 4.8)	20.3 (17.4, 26.2)	-33.4 to 37.9	18.3	13.6 (11.9, 15.4)	58.2	22.2
EKFC$_{cr-cys}$	-5.1 (-7.4, -2.9)	21.0 (16.5, 27.0)	-37.0 to 26.8	17.0	14.0 (12.6, 15.4)	51.3	18.0
**Age < 50 years (n=223)**							
CKD-EPI$_{Cr(2021)}$	-20.6 (-23.6, -17.7)	33.0 (26.0, 38.0)	-64.4 to 23.3	30.4	23.7 (21.1, 26.4)	43.9	12.1
CKD-EPI$_{Cr_Cys(2021)}$	-8.6 (-11.2, -5.9)	24.0 (20.0, 29.0)	-48.8 to 31.7	22.2	16.8 (14.9, 18.7)	57.9	18.8
CKD-EPI$_{Cr(2009)}$	-19.1 (-22.1, -16.2)	32.1 (25.7, 37.7)	-63.0 to 24.9	29.4	22.7 (20.1, 25.4)	44.4	13.9
CKD-EPI$_{Cr-Cys(2012)}$	-2.8 (-5.2, -0.1)	20.4 (17.5, 25.4)	-41.4 to 35.9	19.9	14.8 (13.0, 16.6)	60.5	21.1
CKD-EPI$_{Cys}$	1.8 (-0.9, 4.4)	21.0 (17.1, 25.2)	-37.7 to 41.1	20.1	14.6 (12.9, 16.5)	62.3	23.8
2020$_{Csy-B2M-BTP}$	3.8 (1.0, 6.7)	23.4 (18.0, 27.6)	-36.8 to 44.2	20.9	15.1 (13.2, 17.1)	62.8	18.8

$2020_{Cr\text{-}Csy\text{-}B2M\text{-}BTP}$	-4.8 (-7.3, -2.5)	20.0 (17.0, 23.3)	-40.9 to 31.2	19.0	14.4 (12.9, 16.1)	61.9	22.9
$EKFC_{cr}$	-17.1 (-20.0, -14.4)	28.2 (21.0, 33.4)	-57.2 to 23.1	26.6	20.7 (18.4, 23.0)	48.4	17.0
$EKFC_{cys}$	-2.3 (-5.2, 0.4)	21.1 (16.8, 25.5)	-43.6 to 38.9	21.1	15.1 (13.2, 17.4)	60.5	23.8
$EKFC_{cr\text{-}cys}$	-9.7 (-12.0,-7.4)	21.4 (17.7, 25.5)	-45.3 to 25.9	20.6	16.1 (14.4, 17.8)	55.6	22.4

B2M: β2-Microglobulin, BTP: β-Trace Protein, CI: Confidence interval, CKD: Chronic Kidney Disease, CKD-EPI: Chronic Kidney Disease Epidemiology Collaboration, Cr: Creatinine, Cys: cystatin C, EKFC: European kidney function consortium, eGFR: Estimated glomerular filtration rate, IQR: interquartile range, mGFR: Measured glomerular filtration rate, MAE: mean absolute error, P_{30}: Percentage of participants with eGFR within ±30% of mGFR, P_{10}: Percentage of participants with eGFR within ±10% of mGFR, RMSE: root mean square error.

Bias was expressed as the mean difference in measured GFR minus estimated GFR (95% bootstrapped confidence interval).

Precision was expressed as the interquartile range (IQR) of differences in measured GFR minus estimated GFR (95% bootstrapped confidence interval).

95% distribution of bias was expressed as mean ± 1.96*Standard deviation.

Table S4. Performance of GFR estimating equations as compared to measured GFR in participants with mGFR: ≥45 and <45 ml/min/1.73m²

Method	Mean bias (95%CI) (ml/min/1.73m²)	Precision IQR (95% CI) (ml/min/1.73m²)	Accuracy				
			95% distribution of bias (ml/min/1.73m²)	RMSE (ml/min/1.73m²)	MAE (95% CI) (ml/min/1.73m²)	P_{30} (%)	P_{10} (%)
**Study participants with mGFR ≥45 /min/1.73m² (n=224)**							
CKD-EPI$_{Cr(2021)}$	-23.8 (-26.7, -20.8)	35.9 (32.0, 40.7)	-69.3 to 21.8	33.1	27.6 (25.3, 30.1)	49.1	15.2
CKD-EPI$_{Cr_Cys(2021)}$	-6.7 (-9.8, -3.7)	34.9 (28.4, 39.6)	-52.2 to 38.8	24.1	20.0 (18.1, 21.8)	59.8	20.1
CKD-EPI$_{Cr(2009)}$	-21.4 (-24.2, -18.4)	35.6 (31.9, 41.3)	-67.0 to 24.3	31.6	26.2 (23.8, 28.5)	50.4	19.6
CKD-EPI$_{Cr-Cys(2012)}$	0.9 (-2.1, 3.7)	32.1 (27.6, 36.1)	-43.0 to 44.8	22.4	18.0 (16.2, 19.8)	63.4	23.2
CKD-EPI$_{Cys}$	9.0 (5.9, 11.9)	29.4 (26.0, 33.5)	-36.0 to 53.9	24.6	19.0 (16.8, 21.2)	66.1	25.9
2020$_{Csy-B2M-BTP}$	11.6 (8.7, 14.4)	26.6 (23.1, 30.4)	-31.2 to 54.3	24.6	19.0 (16.9, 21.0)	68.3	22.3
2020$_{Cr-Csy-B2M-BTP}$	-0.5 (-3.2, 2.3)	29.9, 25.3, 33.5)	-41.1 to 40.2	20.7	16.5 (14.7, 18.1)	68.8	28.6
EKFC$_{cr}$	-17.3 (-20.1,-14.5)	32.8 (28.2, 37.8)	-60.5 to 26.0	28.0	22.9 (20.9, 25.0)	56.7	21.4
EKFC$_{cys}$	6.6 (3.7, 9.5)	27.3 (23.9, 30.8)	-36.6 to 49.9	23.0	17.5 (15.4, 19.5)	69.6	26.3
EKFC$_{cr-cys}$	-5.3 (-8.1, -2.6)	30.6 (26.4, 33.6)	-45.1 to 34.4	20.9	17.4 (15.8, 19.0)	67.0	24.6
**Study participants with mGFR < 45ml/min/1.73m² (n=188)**							
CKD-EPI$_{Cr(2021)}$	-13.8 (-16.5,-10.9)	16.0 (12.9, 20.4)	-49.4 to 21.7	22.8	16.7 (14.7, 19.0)	31.9	6.9
CKD-EPI$_{Cr(2009)}$	-11.9 (-14.5, -9.1)	15.3 (12.5, 19.3)	-46.2 to 22.4	21.1	15.4 (13.4, 17.4)	46.30	8.0
CKD-EPI$_{Cr-Cys(2012)}$	-3.0 (-4.7, -1.2)	11.7 (9.8, 14.3)	-25.9 to 20.0	12.1	8.8 (7.6, 10.0)	55.9	18.6
CKD-EPI$_{Cys}$	-2.9 (-4.5, -1.4)	13.7 (11.5, 15.8)	-23.9 to 18.2	11.1	8.5 (7.5, 9.5)	54.8	19.1
2020$_{Csy-B2M-BTP}$	-6.2 (-7.7, -4.8)	12.2 (9.0, 14.4)	-25.6 to 13.1	11.7	9.2 (8.1, 10.2)	54.3	18.6
2020$_{Cr-Csy-B2M-BTP}$	-8.5 (-10.2, -6.7)	12.9 (10.0, 15.7)	-31.4 to 14.4	14.4	11.0 (9.6, 12.2)	46.3	16.0

$EKFC_{cr}$	-12.2 (-14.7, -9.6)	14.4 (12.4, 18.7)	-44.2 to 19.7	20.4	15.1 (13.2, 17.0)	35.1	10.1
$EKFC_{cys}$	-8.4 (-10.3, -6.6)	13.5 (11.2, 15.8)	-33.7 to 17.0	15.4	10.8 (9.2, 12.5)	47.3	19.10
$EKFC_{cr\text{-}cys}$	-10.3 (-12.1,-8.4)	13.3 (10.0, 16.3)	-35.5 to 14.9	16.5	12.5 (10.9, 13.9)	37.8	15.4

B2M: β2-Microglobulin, BTP: β-Trace Protein, CI: Confidence interval, CKD: Chronic Kidney Disease, CKD-EPI: Chronic Kidney Disease Epidemiology Collaboration, Cr: Creatinine, Cys: cystatin C, EKFC: European kidney function consortium, eGFR: Estimated glomerular filtration rate, IQR: interquartile range, mGFR: Measured glomerular filtration rate, MAE: mean absolute error, P_{30}: Percentage of participants with eGFR within ±30% of mGFR, P_{10}: Percentage of participants with eGFR within ±10% of mGFR, RMSE: root mean square error.

Mean bias was expressed as the mean difference in measured GFR minus estimated GFR (95% bootstrapped confidence interval).

Precision was expressed as the interquartile range (IQR) of differences in measured GFR minus estimated GFR (95% bootstrapped confidence interval).

95% distribution of bias was expressed as mean ± 1.96*Standard deviation.
